# Bacterial Nanocellulose Hydrogel for the Green Cleaning of Copper Stains from Marble

**DOI:** 10.3390/gels10020150

**Published:** 2024-02-18

**Authors:** Erica Sonaglia, Emily Schifano, Mohammad Sharbaf, Daniela Uccelletti, Anna Candida Felici, Maria Laura Santarelli

**Affiliations:** 1 Department of Chemical Engineering Materials and Environment, Sapienza University of Rome, Via Eudossiana 18, 00184 Rome, Italy; erica.sonaglia@uniroma1.it (E.S.); mohamadsharbaf@yahoo.com (M.S.); 2 Department of Biology and Biotechnologies “C. Darwin”, Sapienza University of Rome, Piazzale Aldo Moro 5, 00185 Roma, Italy; emily.schifano@uniroma1.it (E.S.); daniela.uccelletti@uniroma1.it (D.U.); 3 Research Center for Nanotechnology Applied to Engineering, Sapienza University of Rome, Piazzale Aldo Moro 5, 00185 Rome, Italy; 4 Department of Basic and Applied Sciences for Engineering, Sapienza University of Rome, Via Antonio Scarpa 16, 00161 Rome, Italy; annacandida.felici@uniroma1.it

**Keywords:** marble, copper stain, hydrogel, cultural heritage, nanocellulose

## Abstract

Cultural heritage stone materials frequently experience significant discoloration induced by copper corrosion products, especially calcareous stones associated with bronze or copper statues and architectural elements. This alteration originates from the corrosion of unprotected copper, resulting in the formation of various Cu minerals and the migration of soluble ions to adjacent stone materials. Traditional cleaning methods involve mechanical, chemical, and laser techniques, which are generally time-consuming, costly, not ecological, or can possibly damage original materials. The loading of highly effective chelating agents, such as ethylenediaminetetraacetic acid (EDTA), into hydrogels has recently been exploited. However, the preference for synthetic hydrogels has been prominent until now, although they lack renewability and biodegradability and require high costs. This study explores for the first time the potential to clean copper corrosion with bacterial nanocellulose (BC) loaded with EDTA as a biologically based, sustainable, and biodegradable hydrogel. The BC hydrogel was characterised by field emission–scanning electron microscopy (FE–SEM), X-ray diffraction analysis (XRD), attenuated total reflectance–Fourier transform infrared spectroscopy (ATR–FTIR), simultaneous thermal analysis (TG-DSC), and tensile testing. It revealed a nano-fibrous structure with high crystallinity and purity and mechanical properties suitable for cultural heritage applications. The EDTA-loaded hydrogel effectively removed copper stains from marble after 120 min of application. Micro-Raman and colorimetric analyses assessed the cleaning efficacy. The study introduces bacterial nanocellulose as a green and effective alternative for heritage conservation, aligning with sustainable methodologies in stone conservation.

## 1. Introduction

The alteration induced by copper corrosion products is a significant challenge in the conservation of architectural and cultural heritage materials. A significant problem related to the presence of corrosion is the staining of calcareous stones such as pedestals or wall facings which are in connection with bronze or copper statues, joints, or architectural elements. The corrosion of unprotected copper, governed by various processes that occur when the metal is exposed to atmospheric conditions, leads to the formation of different Cu minerals. Oxidation by atmospheric oxygen is known to be the first step in this process, forming Cuprite (Cu_2_O) [1]. Successively, moisture, such as rainwater, humidity, or dew, provides the necessary medium for Cu^2+^ ion migration and reaction with other atmospheric compounds, forming a patina [2,3]. It consists mainly of copper (II) sulfates, such as brochantite (Cu_4_(SO_4_)(OH)_6_) and posnjakite (Cu_4_SO_4_(OH)_6_·H_2_O), and/or chlorides like atacamite (Cu_2_Cl(OH)_3_) [1,4]. In some cases, carbonates such as malachite (Cu_2_(CO_3_)(OH)_2_) or azurite (Cu_3_(CO_3_)_2_(OH)_2_) are also found [5]. Soluble ions can therefore migrate from the metal superficial patina to adjacent stone materials thanks to water action [6], where their reprecipitation occurs, resulting in significant discoloration (Figure S1).

Traditional methods for cleaning stone surfaces of metal corrosion products involves mechanical techniques, chemical treatments, and laser ablation. Among chemical cleaning methods, chelating agents are commonly preferred over acidic or alkaline solutions and reducing agents because of their higher efficacy [2,7]. One of the most widely used chelating agents in heritage conservation is ethylenediaminetetraacetic acid (EDTA). Generally, it forms stable complexes with ions, promoting their removal from the stone surface [8].

Several studies have suggested that the incorporation of EDTA into hydrogel systems can be promising [7,9,10]. Indeed, the hydrogels offer a controlled and localised delivery of EDTA, allowing for a targeted application and prolonged contact with the corrosion products, minimising any negative impact on the heritage material [11]. Furthermore, the use of hydrogels facilitates a more sustainable and environmentally friendly approach, as they minimise leaching and require a smaller amount of product, also ensuring long-term economic savings [9]. Since the introduction of gels in the cultural heritage field, synthetic polymer-derived gels have shown greater versatility and superior performance compared to naturally based gels [10,12]. However, given their acknowledged non-renewability and frequent non-biodegradability, there is a growing need for sustainable alternatives [13]. Ongoing research is now facing these limitations, focusing on the development of new natural polymer-based hydrogel formulations by studying advanced crosslinking methods [14] and novel natural polymers [15,16].

Addressing the imperative for environmentally conscious practices, our research aims to introduce and assess the potential of an EDTA-loaded bacterial nanocellulose hydrogel. Bacterial nanocellulose is a biologic material produced by different genera of bacteria as an extracellular polysaccharide, and it can be a by-product of fermented beverages [17]. It is a cost-effective, sustainable, renewable, compatible, and biodegradable material. Indeed, it could represent a green and effective alternative to traditional hydrogels for the cleaning of cultural heritage materials, and its investigation represents a novelty in this field.

## 2. Results and Discussion

### 2.1. BC Hydrogel Characterisation

#### 2.1.1. Microstructure

**Figure 1:**
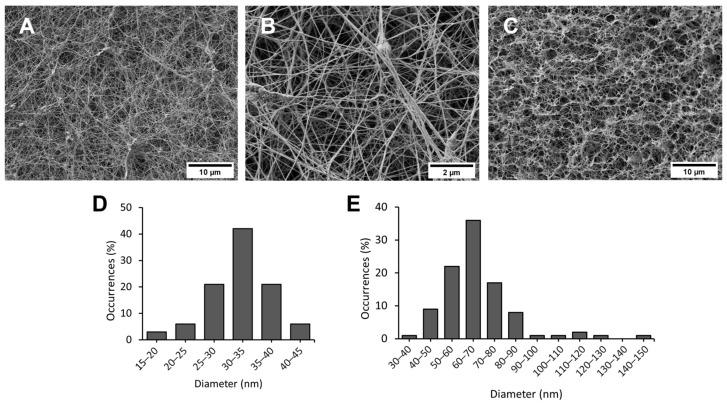
Microstructural analysis of the BC hydrogel. (**A**) SEM micrograph of the sample surface at 5k× magnification; (**B**) SEM micrograph of the sample surface at 25k× magnification; (**C**) SEM micrograph of the the cross-section at 5k× magnification; (**D**) Number of occurrences (%) of fibril diameters; (**E**) Number of occurrences (%) of ribbon diameters.

The BC microstructure was investigated by SEM to assess its potential as a hydrogel material. The micrographs taken at different magnifications showed a network of interconnected cellulose fibrils that form some aggregates (ribbons). The sample surface displays an expanded network with high and open porosity with randomly oriented fibrils (Figure 1A,B). Cross-section analysis reveals a complex architecture with a diffuse porosity (Figure 1C). The architecture of the investigated material classifies the BC as a nano-fibrous hydrogel [18]. Moreover, these results are in agreement with the morphological descriptions of BC obtained from various bacterial strains [19,20]. Dimensional analysis results (Figure 1D,E) indicate that structures exhibit the most frequent diameter values in the ranges of 30–35 nm and 60–70 nm for fibrils and ribbons, respectively. These results are in accordance with data obtained in other studies [21,22].

#### 2.1.2. Crystallinity and Chemical Structure

**Figure 2:**
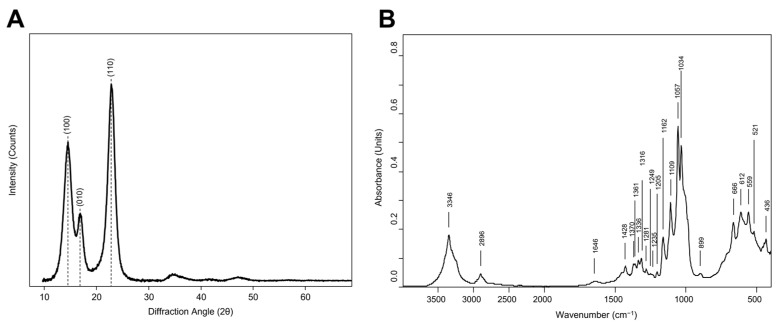
Characterisation analysis of BC hydrogel. (**A**) X-ray diffraction with Miller indices of cellulose Iα crystallographic planes; (**B**) ATR-FTIR spectra.

Sample crystallinity was investigated by XRD analysis. The X-ray diffractogram in Figure 2A reveals three distinct peaks at approximately 2θ = 14, 16, and 22. The diffraction pattern describes a type I cellulose, one of the four crystalline polymorphs of cellulose and a constituent of native cellulose [23]. The primary peaks can be attributed to both the Iα unit cell (Miller indices of (100), (010), and (110)) and Iβ cell (($1\bar{1}0$), (110), and (200) indices), characteristic of the triclinic and monoclinic systems, respectively. The relative position of the 2θ = 14 and 2θ = 16 peaks indicates the abundance of the two allomorphs [24], with a positive Z index (38.0) suggesting a prevalence of the Iα allomorph, as reported in Section 4. The crystallite size calculated for the main peak is 5.6 nm, in accordance with the literature [21,25]. The calculated Segal crystallinity index (Cr.I.) is 93.7%, showing a high ratio between crystalline and amorphous phases [26]. The results highlight that the nature of cellulose crosslinking in the BC hydrogel is primarily attributed to the interaction of cellulose chains with the formation of diffuse crystalline domains, achieving a unique crosslinking useful for their employment as hydrogels.

The chemical nature of BC hydrogel was evaluated by ATR–FTIR spectroscopy. The IR spectrum (Figure 2B) shows the typical absorption features of pure cellulose. At 4000–2500 cm^−1^, the spectrum shows two large bands. The first one at 3600–3000 cm^−1^ is related to inter- and intra- molecular hydrogen bonds of the O–H group stretching vibrations of cellulose [27]. The second one, at 3000–2800 cm^−1^, was due to the stretching modes of C–H in methyl and methylene groups. The signal at 1646 cm^−1^ is due to the O–H bending [27]. At 1500–1200 cm^−1^, different signals are present due to several bends in CH_2_ and CH bonds (1428 cm^−1^, 1370 cm^−1^, 1361 cm^−1^, 1336 cm^−1^, 1316 cm^−1^, 1316 cm^−1^, 1281 cm^−1^, 1235 cm^−1^) [22,28,29]. At 1200–800 cm^−1^. the absorptions of C–O–C and C–OH groups are present at 1057 cm^−1^ and 1034 cm^−1^, referring to stretching vibrations of carbohydrate macromolecules and side groups [29,30]. The typical cellulose Iα signals at 3240 cm^−1^ and 750 cm^−1^ and Iβ absorptions at 3270 cm^−1^ and 710 cm^−1^ are present [31]. These results are in accordance with the XRD observations, confirming the presence of the two crystalline allomorphs of cellulose I. The advantages of using a pure cellulose hydrogel lie primarily in the cellulose insolubility in many common solvents due to the existence of extensive intra- and inter-molecular hydrogen bonding [32]. A pure cellulose hydrogel would maintain its structural integrity and effectiveness without being compromised by the solvents used in the treatment processes.

#### 2.1.3. Tensile and Thermal Behaviours

**Figure 3:**
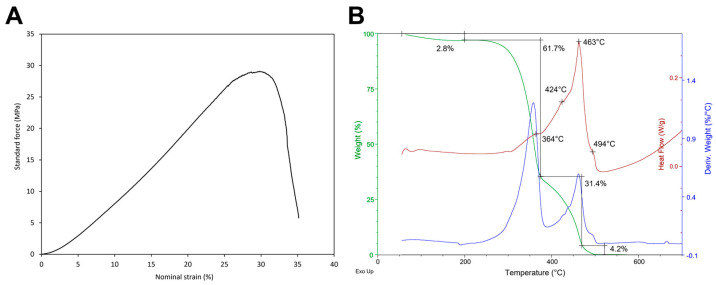
Tensile and thermal behaviours of BC hydrogel. (**A**) Tensile stress (MPa)–strain (%) curve of BC hydrogel; (**B**) Simultaneous thermal analysis (TG-DSC) of BC hydrogel (green—TG curve, blue—DTG curve, red—DSC curve).

Tensile testing was used to evaluate the mechanical properties of the BC hydrogel. Figure 3A shows the obtained stress (MPa)–strain (%) plot. The curve displayed a viscoelastic behaviour, where the profile was influenced by the squeezing out of water during the test [33,34,35]. The maximum percentage of elongation, ε, was 28.49 ± 1.76%, the maximum yield stress, σ, was 26.73 ± 1.77 MPa, and the elastic modulus, E, was 1.16 ± 0.06 MPa. The results underline the prevalence of elastic behaviour upon plastic behaviour, which derives from the stretching of cellulose fibrils until their tearing and breaking point, as also attested by other authors [35,36]. The highlighted mechanical behaviour shows a viscoelastic material with high tensile strength, which is suitable for several kinds of applications.

A thermogravimetric analysis of BC hydrogel was performed and is reported in Figure 3B. An initial weight loss (2.8%) was observed up to 200 °C, which is attributed to water evaporation. Subsequently, a consistent weight loss (61.7%) occurred at 364 °C, corresponding to the dehydration of cellulose [25]. A large exothermic effect in DSC (weight loss of 31.4%) was observed in the range 400–500 °C, with main peaks at 424 °C and 463 °C involving the decomposition, oxidation, and depolymerisation of glycoside units of cellulose. At 494 °C, weight loss (4.2%) due to the breakdown of carbonaceous residues yielding low molecular weight gaseous products is observed [25]. This thermal behaviour aligns with the literature reports on BC [37].

#### 2.1.4. Water Holding Capacity (WHC) and Water Release Rate (WRR)

The WHC represents the ratio between the retained water and the dry weight of cellulose, and it is a characteristic property of the hydrogel [15,38,39,40,41]. The evaluated WHC for the BC hydrogel is 67.0 ± 2.4, comparable with the range of values reported for BCs produced by different strains and culture media [42]. The result confirms the presence of a high quantity of water due to its interaction with numerous free hydroxyl groups present in cellulose fibrils, creating hydrogen bonds with water [43]. The WRR represents the grams of evaporated water per time and assesses the hydrogel’s capacity to retain the liquid phase. The evaluated WRR resulted in 0.302 mg/min. Even though it possesses a very high WHC, this data underscores the strong water retention of the BC network, which is comparable with results obtained by various groups concerning hydrogel for cultural heritage cleaning [16,44]. Specifically, the characterisation highlighted a hydrogel with a high WHC that can be employed for prolonged treatment durations based on its low WRR.

### 2.2. EDTA-Loaded BC Hydrogel and Cleaning Efficiency Evaluation

**Figure 4:**
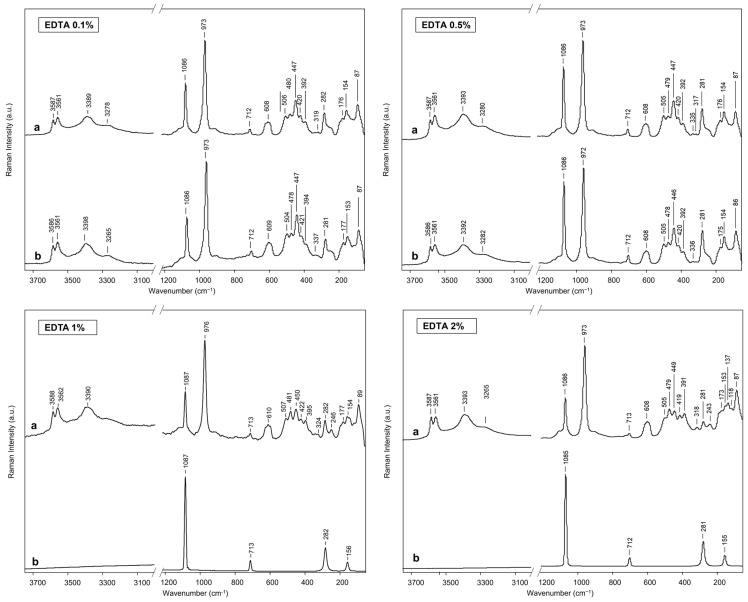
Raman spectra for cleaning efficacy evaluation of the tested concentrations (EDTA 0.1% *w*/*v*, EDTA 0.5% *w*/*v*, EDTA 1% *w*/*v*, EDTA 2% *w*/*v*). (a) Stained specimen. (b) Specimen treated with EDTA-loaded BC hydrogels for 120 min.

For testing the cleaning efficiency of the EDTA-loaded BC hydrogels at various concentrations, an experimental simulation of cleaning marble stained with Cu-based stains was carried out. BC hydrogels were loaded with 0.1% *w*/*v*, 0.5% *w*/*v*, 1% *w*/*v*, and 2% *w*/*v* aqueous solutions of disodium EDTA, as described in Section 4. The cleaning process was executed by applying the EDTA-loaded BC hydrogels to artificially stained marble specimens for 120 min in comparison to the treatment with the pristine BC hydrogel. To assess the removal of copper stains, µ-Raman analysis was performed on the areas before and after the treatment (Figure 4). For all areas, the spectra collected before cleaning show signals at 1086 cm^−1^, 713 cm^−1^, 282 cm^−1^, and 154 cm^−1^ assigned to the calcite mineral of marble and strong signals associated to Cu sulfate minerals, in which brochantite can be detected, as reported in [45,46]. The spectra collected for the areas treated with 0.1% *w*/*v* and 0.5% *w*/*v* EDTA-loaded BC hydrogels still present extensive signals which are related to the Cu staining layer, demonstrating the inefficacy of the two tested concentrations (Figure 4). In contrast, the spectra collected for the areas treated with 1% *w*/*v* and 2% *w*/*v* EDTA-loaded BC hydrogels show only signals referred to calcite, highlighting the effectiveness of the cleaning treatment. The results are in agreement with other studies in which a 1% *w*/*v* EDTA concentration was found effective in agar gels [3,7]. However, the BC hydrogels do not require heating for preparation. Moreover, Raman spectra obtained after the cleaning show no presence of cellulose signals, typically centered at 2900 cm^−1^ and 1095 cm^−1^ [47]. The results highlight the absence of polymeric residues on treated surfaces, as already assessed for the self-standing peelable gels in [48] (Figure S2).

**Figure 5:**
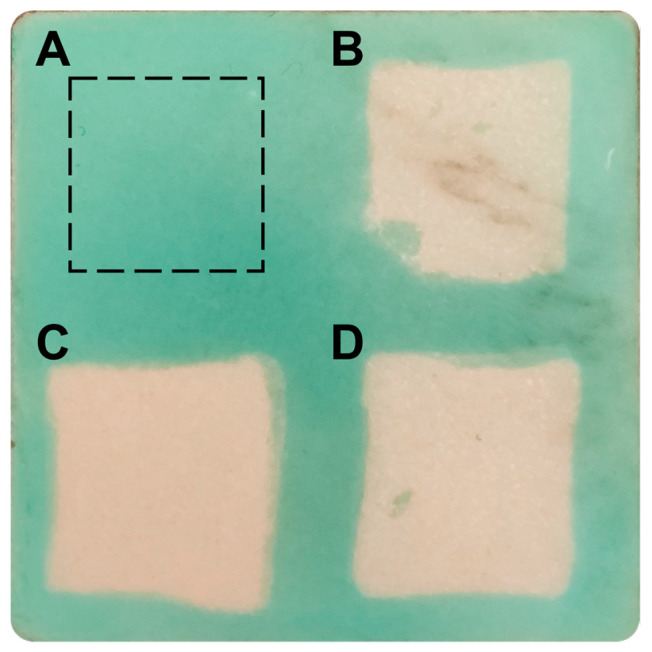
Photograph of the stained marble specimen after the cleaning treatments. (**A**) Negative control area treated with pristine BC hydrogel for 120 min. (**B**) Area treated with 1% *w*/*v* EDTA-loaded BC hydrogel for 30 min. (**C**) Positive control area treated with 1% *w*/*v* EDTA-loaded BC hydrogel for 120 min. (**D**) Area treated with 1% *w*/*v* EDTA-loaded BC hydrogel for 60 min.

To assess the influence of application time on cleaning efficacy, the 1% *w*/*v* EDTA-loaded BC hydrogels were applied to stained marble for 30 and 60 min. The pristine BC hydrogel was applied as the negative control, while the 1% *w*/*v* EDTA-loaded BC hydrogel applied for 120 min served as the positive control (Figure 5). The µ-Raman analysis on the areas after cleaning demonstrated that the areas treated for 30 and 60 min still presented some Cu residues in evenly distributed spots (Figures S3 and S4).

**Figure 6:**
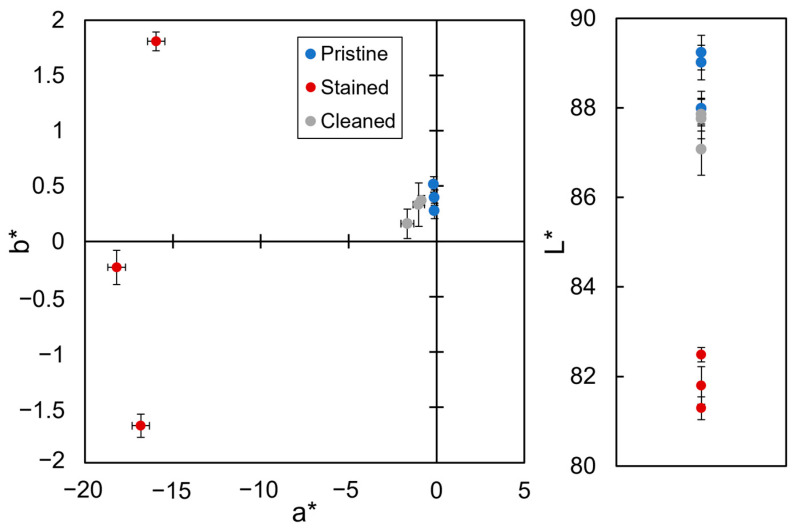
Colorimetric analysis. Chromatic parameters (L*, a*, b*) obtained for selected areas of laboratory specimens in their pristine state (blue, pristine), after staining (red, stained), and after the cleaning treatment with 1% *w*/*v* EDTA-loaded BC hydrogels for 120 min (gray, cleaned).

Colorimetric analysis was performed to further assess the cleaning efficacy of the 1% *w*/*v* EDTA-loaded BC hydrogel applied for 120 min, as it emerged from previous analysis as the most effective hydrogel. The L*, a*, and b* values in the CIELab colour system for pristine, stained, and cleaned specimens were obtained. Results are reported in Table 1. In the colour system, the coordinates L*, a*, and b* are the brightness, the red–green component, and the yellow–blue component, respectively [49]. Pristine specimens present a white colour with high brightness. Small value deviations confirm the colour homogeneity of the specimens which derive from the same marble batch. When stained, the specimens show a marked increase in green and blue components with a decrease in brightness. Values of a* and b* show significant colour deviation, reflecting some inhomogeneity in the staining layers, which was also attested in cultural heritage materials with naturally formed copper stains [7]. Instead, the chromatic values of cleaned specimens fall in the same range as the values of pristine specimens, as also reported in Figure 6. The principal component analysis (PCA) is indeed able to cluster the stained specimens far from the pristine and cleaned samples, which are clustered close together. The data agree with µ-Raman spectroscopy results, confirming the cleaning by the EDTA-loaded BC hydrogel. Furthermore, to evaluate the efficacy of the cleaning treatment, the ΔE* value, referring to the chromatic parameters of pristine and cleaned specimens, was calculated. Indeed, according to García and Malaga [50] and Ortiz et al. [51] the human perception of colour can be divided into three different ranges: ΔE*  <  5, chromatic that changes cannot be detected by the human eye; 5  <  ΔE* <  10, chromatic changes that can be detected by the human eye; and ΔE*  >  10, chromatic changes that are clearly visible. Generally, cleaning treatments on cultural heritage stones that generate ΔE*  <  5 are accepted as effective [52]. In this study, the calculated ΔE* is 1.61 ± 0.42, indicating the high cleaning efficacy of the 1% *w*/*v* EDTA-loaded BC hydrogel applied for 120 min.

The proposed system could potentially serve as a feasible alternative to the current techniques used for removing copper stains. While hydrogels loaded with chelating agents have shown success, synthetic polymers and chemical crosslinkers are not eco-friendly and have high production costs. Some issues are also present when dealing with hydrogels based on natural polymers, mainly polysaccharides such as agar, gellan gum, and xanthan gum, in physically crosslinked formulations [13,53]. Agar hydrogels are rigid, making it difficult to achieve optimal performance on rough substrates, and they can be fragile. Additionally, the need to undergo heating poses logistical challenges. Moreover, the growing use of agar raises concerns about its availability [13]. Gellan and xanthan gum hydrogels may exhibit limited compatibility with certain complexing agents or solutions [53]. Furthermore, the high viscosity complicates the removal, leading to the presence of residues on treated surfaces.

In contrast to conventional hydrogels, BC offers significant advantages due to its composition and naturally crosslinked fibril network. Indeed, BC hydrogels are produced through a safe biological process and do not involve the use of cross-linkers. In terms of application, BC hydrogels are self-supporting and easily shaped, enabling precise targeting of the solvent action, making them particularly suitable for treating multi-material artifacts. Furthermore, they are easily removable (peelable) and leave no residues on surfaces due to the strong crosslinking of cellulose fibrils, which show diffuse crystalline domains. BC hydrogels require no additional modifications, such as thermal treatments. Moreover, they are not rigid and show viscoelastic properties suitable for adhering to rough and vertical surfaces; furthermore, they can be loaded with organic solvents.

## 3. Conclusions

In this study, the potential of bacterial nanocellulose (BC) hydrogel loaded with ethylenediaminetetraacetic acid (EDTA) for the green cleaning of copper stains from marble has been exploited. The characterisation of the BC hydrogel, including its microstructure, crystallinity, chemical structure, thermal and tensile behaviours, water holding capacity, and water release rate, confirmed its suitability as a hydrogel material for cultural heritage applications. The removal of copper stains from marble specimens was evaluated for different EDTA concentrations and treatment times. The cleaning efficacy was assessed by µ-Raman spectroscopy and colorimetric analysis, demonstrating complete Cu stain removal after the treatment with 1% *w*/*v* EDTA-loaded hydrogel for 120 min. BC hydrogels offer several advantages over conventional hydrogels, including a naturally crosslinked fibril network, ease of shaping, and self-supporting properties. Moreover, BC hydrogels do not require additional modifications such as thermal treatments and are easily removable, leaving no residues on treated surfaces. The sustainable nature of BC hydrogels, combined with their effectiveness in stain removal and compatibility with heritage materials, makes them a promising alternative to traditional cleaning methods. This study paves the way for further exploration and development of natural polymer-based hydrogel formulations for heritage conservation.

## 4. Materials and Methods

### 4.1. Bacterial Nanocellulose (BC) Hydrogel Characterisation

#### 4.1.1. BC Purification

Bacterial nanocellulose (BC) hydrogel was supplied by a brewer located in Duino Aurisina (TS), Italy. Samples of approximately 10 cm × 10 cm were purified in an aqueous solution of 0.5 M NaOH (Merck, Darmstadt, Germany) at 85 °C for 1 h three times. Afterward, the samples were soaked in H_2_O_dd_ at room temperature overnight, then were rinsed repeatedly until the samples reached a neutral pH. Samples were stored in H_2_O_dd_ at 4 °C until further use.

#### 4.1.2. BC Microstructure Analysis

To characterize the microstructure, samples of BC hydrogel (dimension 2 cm × 2 cm) were treated using the freeze-drying method. Indeed, samples were immersed in liquid nitrogen and placed in a Lyovapor L-200 instrument (Buchi, Flawil, Switzerland) for 24 h. The microstructure was visualised using an FE-SEM Zeiss Auriga 405 (Zeiss, Oberkochen, Germany). Micrographs from surface and cross-section samples were collected at 1.20 keV. Fibrils and fibril ribbon diameter distributions were evaluated using ImageJ 1.53k Java 1.8.0_172 software for 100 measurements each.

#### 4.1.3. BC Crystallinity Analysis

X-ray diffraction analysis was performed using a D8 Advance diffractometer (Bruker AXS Gmbh, Ettlingen, Germany). The diffractometer operates in Bragg–Brentano geometry with Mo–Ka radiation (λ = 0.7093 Å, 40 keV, 35 mA). Angular scanning was carried out between 3.3° and 29.98° with a step of 0.01°. We used a home-made “fork-type” sample stage to minimize the background. X-ray diffraction patterns were converted to the CuKα1 (λ = 1.5418 Å) wavelength for easier comparison with the literature data.

The crystallinity index (CrI) (%) was calculated for comparison purposes according to the method proposed by Segal, Creely, Martin Jr., and Conrad (1959) [26] using the maximum intensity of the diffraction peak of the crystalline region (I_200_) and the minimum intensity of the amorphous region (I_am_) at an angle of 2θ∼18°, as shown in Equation (1).

(1) $$\mathrm{Cr}.I.=\frac{\left(I_{200}-I_{\mathrm{am}}\right)}{I_{200}}\times 100,$$

The interplanar spacing (d) was calculated by means of Bragg’s law (Equation (2)):

(2) $$n\lambda =2d\;\mathrm{sen}\left(\theta \right),$$

where n (integer) is the order of reflection, λ is the wavelength of the incident X-rays, d is the interplanar spacing of the crystal, and θ is the angle of incidence.

The average crystallite size (L) (nm) along the three cell planes related to the detected peaks was calculated using the Scherrer formula (Equation (3)):

(3) $$L=\frac{K_{s}\lambda }{\tau \cos\theta },$$

where K_s_ is a shape factor constant in the range 0.8–1.2 (typically equal to 0.9), λ is the X-ray wavelength, τ is the peak full width at half maximum (FWHM), and θ is the Bragg angle. Crystal allomorph abundance (Iα and Iβ) was estimated by Equation (4), developed by Wada and co-workers [24]:

(4) $$Z=1693\;d_{1}-902d_{2}-549,$$

where d_1_ (nm) is the d-spacing of (100) of Iα and ($1\bar{1}0$) of Iβ plane and d_2_ (nm) is the d-spacing of (010) of Iα and (110) of Iβ planes. The Z value is >0 for Iα-rich cellulose and <0 for Iβ-rich cellulose.

#### 4.1.4. BC Chemical Structure Analysis

Attenuated total reflectance–Fourier transform infrared spectroscopy (ATR–FTIR) spectra were acquired to determine the chemical structure of the BC hydrogel. The analysis was executed using a Vertex 70 spectrometer (Bruker Optics, Gmbh, Ettlingen, Germany) equipped with a single reflection Diamond ATR cell, a standard MIR source (HeNe), and a room temperature DTGS detector. The spectra were recorded with 64 scans in the mid-infrared range (400–4000 cm^−1^) at a resolution of 4 cm^−1^.

#### 4.1.5. BC Water Holding Capacity (WHC)

The WHC was estimated according to Equation (5), as reported in [41]:

(5) $$\mathrm{WHC}=\frac{\left(M_{\mathrm{wet}}-M_{\mathrm{dry}}\right)}{M_{\mathrm{dry}}},$$

where M_wet_ is the mass of the sample after the careful removal of excess water from the sample surface by tapping it with a tissue and M_dry_ is the mass of the sample after oven-drying at 60 °C until no weight change is detected. Measurements were conducted in quadruplicate.

#### 4.1.6. BC Water Release Rate (WRR)

The WRR of the BC hydrogel was estimated by an ad hoc experiment performed with an SDT-Q600 (TA Instruments, New Castle, DE, USA) analyzer. Measurements were made on approximately 80 mg of sample pre-conditioned in a 100% relative humidity atmosphere cell for 24 h. Sample weight was monitored in a dehydration experiment in isothermal conditions at 40 °C under a nitrogen flow rate of 20 mL/min. The WRR was estimated for the weight vs. time (mg/min) plots using TA Universal Analysis 2000 software (version 4.5A), and it is equal to the slope of the graph in the linear trait of the curve.

#### 4.1.7. BC Thermal Behaviour

Samples were heated under an artificial air flow rate of 100 mL/min with a 10 °C/min heating rate until 1000 °C using the same instrument as described before. The thermal behaviour was analysed by plotting the weight (%) vs. temperature (°C) curve. Differential thermal gravimetry (DTG) and differential scanning calorimetry (DSC) curves were still plotted. The weight loss and the peak temperatures were evaluated in the TG curve and the DSC curve, respectively.

#### 4.1.8. BC Tensile Behaviour

Uniaxial elongation studies were performed on BC hydrogel according to [54]. Briefly, the hydrogels having a rectangular shape of 45 mm × 15 mm were fixed in an Instron 5982 instrument (Instron, Milan, Italy) with a 1 kN load cell. The samples were elongated at a rate of 1 mm/min until breaking point at room temperature. The maximum percentage of elongation, ε, the maximum yield stress, σ, and the elastic modulus, E, were determined, and the stress–strain curve was plotted.

### 4.2. BC Hydrogel Loading with EDTA Solution

To achieve hydrogel loading with chelating agent solution, gel samples (1.5 cm × 1.5 cm) underwent solvent exchange. Briefly, samples were immersed in disodium EDTA (C_10_H_14_N_2_Na_2_O_6_·2H_2_O, Merck-Millipore, Burlington, MA, USA) aqueous solution (0.1% *w*/*v*, 0.1% *w*/*v*, 1% *w*/*v*, and 2% *w*/*v*), and the solvent was exchanged every 24 h three times at room temperature.

### 4.3. Preparation of Laboratory Specimens

Homogeneous distributed copper stains were obtained on Carrara marble specimens (5 cm × 5 cm × 2 cm) by in situ synthesis of brochantite according to the method proposed by Bakhtiari and Darezereshki (2011) [55]. Marble specimens were immersed in a 0.05 M copper sulfate pentahydrate (CuSO_4_·5H_2_O, Merck-Millipore, Burlington, MA, USA) aqueous solution at 55 °C. Afterward, drops of a 0.05 M sodium carbonate (Na_2_CO_3_, Merck-Millipore, Burlington, MA, USA) aqueous solution were added until the presence of a coloured precipitate. Specimens remained immersed overnight in the reaction solution and were successively air-dried, and a coloured surface was observed on the samples.

### 4.4. Evaluation of the Cleaning Efficacy

For the removal of copper stains from specimens previously described, areas of 0.5 cm × 0.5 cm were selected. To select the best concentration, EDTA-loaded BC hydrogels at different concentrations and times were then applied to the selected specimen areas. Afterward, a transparent film was placed on each hydrogel to minimise water evaporation; the samples were treated for 120 min. As a comparison, an area was treated for 120 min with a water-loaded BC hydrogel. Then, the hydrogels were peeled off and replaced for 30 min with water-loaded BC hydrogels to remove any residues of chelating solution. µ-Raman spectroscopy was applied to the different areas before and after the cleaning treatment with a Senterra spectrometer (Bruker Optics, Gmbh, Ettlingen, Germany) equipped with a CW diode-pumped solid-state laser emitting at 532 nm. The spectral resolution was 9 cm^−1^, and the time of acquisition and number of scans were selected to reduce the fluorescence interferences. In each area, five spectra were acquired at random spots. Colorimetric measurements were performed on specimens with an AvaSpec spectrophotometer (Avantes, Apeldoorn, The Netherlands) equipped with an Avantes HL-2000 FHSA halogen lamp, according to [56]. For each specimen, five measurements were conducted on the same area of dimension 1 cm × 1 cm before staining, after staining, and after the cleaning treatment. The obtained coordinates L*, a*, and b* in the CIELab colorspace describe the lightness, the red–green, and the yellow–blue components, respectively. The ΔE value referring to the average values of the cleaned samples compared to the pristine ones was calculated according to Equation (6).

(6) $$\Delta E=\sqrt{{\left({\Delta L}^{*}\right)}^{2}+{\left({\Delta a}^{*}\right)}^{2}+{\left({\Delta b}^{*}\right)}^{2}},$$

## Figures and Tables

**Table 1:** Average chromatic coordinates in the CIELab colour system of pristine, stained, and cleaned specimens.

	Pristine	Stained	Cleaned
L*	88.75 ± 0.67	81.86 ± 0.60	87.56 ± 0.43
a*	−0.12 ± 0.02	−16.99 ± 1.13	−1.18 ± 0.42
b*	0.40 ± 0.12	−0.03 ± 1.75	0.29 ± 0.11

## Data Availability

The data presented in this study are openly available in article.

## Supplementary Materials

The following supporting information can be downloaded at: https://www.mdpi.com/article/10.3390/gels10020150/s1, Figure S1: Image of copper stains on marble pedestal of a bronze statue (M. Sharbaf); Figure S2: (A) Raman spectrum obtained for the specimen treated with 1% *w*/*v* EDTA-loaded BC hydrogel for 120 min, (B) Raman spectrum obtained for the specimen treated with 2% *w*/*v* EDTA-loaded BC hydrogel for 120 min, (C) Raman spectrum obtained for dry pristine BC hydrogel; Figure S3: Raman spectra of the cleaning efficacy evaluation. (a) Stained specimen. (b) Specimen treated with 1% *w*/*v* EDTA-loaded BC hydrogel for 30 min; Figure S4: Raman spectra of the cleaning efficacy evaluation. (a) Stained specimen. (b) Specimen treated with 1% *w*/*v* EDTA-loaded BC hydrogel for 60 min.

## References

[1] FitzGerald K.P., Nairn J., Skennerton G., Atrens A. (2006). Atmospheric Corrosion of Copper and the Colour, Structure and Composition of Natural Patinas on Copper. Corros. Sci..

[2] Andrea Macchia L.C., Colasanti I.A., Rivaroli L., Favero G., de Caro T., Munoz L.P., Russa M.F.L. (2023). Natural Based Products for Cleaning Copper and Copper Alloys Artefacts. Nat. Prod. Res..

[3] Bertasa M., Canevali C., Sansonetti A., Lazzari M., Malandrino M., Simonutti R., Scalarone D. (2021). An In-Depth Study on the Agar Gel Effectiveness for Built Heritage Cleaning. J. Cult. Herit..

[4] Leygraf C., Chang T., Herting G., Wallinder I.O. (2019). The Origin and Evolution of Copper Patina Colour. Corros. Sci..

[5] Manti P., Watkinson D. (2022). Corrosion Phenomena and Patina on Archaeological Low-Tin Wrought Bronzes: New Data. J. Cult. Herit..

[6] Gaylarde C.C., Gaylarde P.M., Beech I.B. (2008). Deterioration of Limestone Structures Associated with Copper Staining. Int. Biodeterior. Biodegrad..

[7] Canevali C., Fasoli M., Bertasa M., Botteon A., Colombo A., Tullio V.D., Capitani D., Proietti N., Scalarone D., Sansonetti A. (2016). A Multi-Analytical Approach for the Study of Copper Stain Removal by Agar Gels. Microchem. J..

[8] Sansonetti A., Bertasa M., Corti C., Rampazzi L., Monticelli D., Scalarone D., Sassella A., Canevali C. (2021). Optimization of Copper Stain Removal from Marble through the Formation of Cu (II) Complexes in Agar Gels. Gels.

[9] Boccalon E., Nocchetti M., Pica M., Romani A., Sterflinger K. (2021). Hydrogels: A ‘Stepping Stone’ towards New Cleaning Strategies for Biodeteriorated Surfaces. J. Cult. Herit..

[10] Domingues J.A.L., Bonelli N., Giorgi R., Fratini E., Gorel F., Baglioni P. (2013). Innovative Hydrogels Based on Semi-Interpenetrating p(HEMA)/PVP Networks for the Cleaning of Water-Sensitive Cultural Heritage Artifacts. Langmuir ACS J. Surf. Colloids.

[11] Gabriele F., Vetrano A., Bruno L., Casieri C., Germani R., Rugnini L., Spreti N. (2021). New Oxidative Alginate-Biocide Hydrogels against Stone Biodeterioration. Int. Biodeterior. Biodegrad..

[12] Bonelli N., Poggi G., Chelazzi D., Giorgi R., Baglioni P. (2019). Poly(Vinyl Alcohol)/Poly(Vinyl Pyrrolidone) Hydrogels for the Cleaning of Art. J. Colloid Interface Sci..

[13] Passaretti A., Cuvillier L., Sciutto G., Guilminot E., Joseph E. (2021). Biologically Derived Gels for the Cleaning of Historical and Artistic Metal Heritage. Appl. Sci..

[14] Cuvillier L., Passaretti A., Guilminot E., Joseph E. (2024). Agar and Chitosan Hydrogels’ Design for Metal-Uptaking Treatments. Gels.

[15] Prati S., Volpi F., Fontana R., Galletti P., Giorgini L., Mazzeo R., Mazzocchetti L., Samorì C., Sciutto G., Tagliavini E. (2018). Sustainability in Art Conservation: A Novel Bio-Based Organogel for the Cleaning of Water Sensitive Works of Art. Pure Appl. Chem..

[16] Samorì C., Galletti P., Giorgini L., Mazzeo R., Mazzocchetti L., Prati S., Sciutto G., Volpi F., Tagliavini E. (2016). The Green Attitude in Art Conservation: Polyhydroxybutyrate–Based Gels for the Cleaning of Oil Paintings. ChemistrySelect.

[17] Gorgieva S., Trček J. (2019). Bacterial Cellulose: Production, Modification and Perspectives in Biomedical Applications. Nanomaterials.

[18] Přádnỳ M., Šlouf M., Martinová L., Michálek J. (2010). Macroporous Hydrogels Based on 2-Hydroxyethyl Methacrylate. Part 7: Methods of Preparation and Comparison of Resulting Physical Properties. E-Polymers.

[19] Gao X., Shi Z., Kuśmierczyk P., Liu C., Yang G., Sevostianov I., Silberschmidt V.V. (2016). Time-Dependent Rheological Behaviour of Bacterial Cellulose Hydrogel. Mater. Sci. Eng. C.

[20] Hu Y., Catchmark J.M., Zhu Y., Abidi N., Zhou X., Wang J., Liang N. (2014). Engineering of Porous Bacterial Cellulose toward Human Fibroblasts Ingrowth for Tissue Engineering. J. Mater. Res..

[21] Tsouko E., Kourmentza C., Ladakis D., Kopsahelis N., Mandala I., Papanikolaou S., Paloukis F., Alves V., Koutinas A. (2015). Bacterial Cellulose Production from Industrial Waste and By-Product Streams. Int. J. Mol. Sci..

[22] Kiziltas E.E., Kiziltas A., Gardner D.J. (2015). Synthesis of Bacterial Cellulose Using Hot Water Extracted Wood Sugars. Carbohydr. Polym..

[23] French A.D. (2014). Idealized Powder Diffraction Patterns for Cellulose Polymorphs. Cellulose.

[24] Wada M., Okano T. (2001). Localization of Iα and Iβ Phases in Algal Cellulose Revealed by Acid Treatments. Cellulose.

[25] Vasconcelos N.F., Feitosa J.P.A., da Gama F.M.P., Morais J.P.S., Andrade F.K., Filho M.d.S.M.d.S., de Rosa M.F. (2017). Bacterial Cellulose Nanocrystals Produced under Different Hydrolysis Conditions: Properties and Morphological Features. Carbohydr. Polym..

[26] Segal L., Creely J.J., Martin Jr A., Conrad C. (1959). An Empirical Method for Estimating the Degree of Crystallinity of Native Cellulose Using the X-Ray Diffractometer. Text. Res. J..

[27] Cichosz S., Masek A. (2020). IR Study on Cellulose with the Varied Moisture Contents: Insight into the Supramolecular Structure. Materials.

[28] Nelson M.L., O’Connor R.T. (1964). Relation of Certain Infrared Bands to Cellulose Crystallinity and Crystal Latticed Type. Part I. Spectra of Lattice Types I, II, III and of Amorphous Cellulose. J. Appl. Polym. Sci..

[29] Higgins H., Stewart C., Harrington K. (1961). Infrared Spectra of Cellulose and Related Polysaccharides. J. Polym. Sci..

[30] Hong T., Yin J.-Y., Nie S.-P., Xie M.-Y. (2021). Applications of Infrared Spectroscopy in Polysaccharide Structural Analysis: Progress, Challenge and Perspective. Food Chem. X.

[31] Sugiyama J., Persson J., Chanzy H. (1991). Combined Infrared and Electron Diffraction Study of the Polymorphism of Native Celluloses. Macromolecules.

[32] Mohd N., Draman S.F.S., Salleh M.S.N., Yusof N.B. (2017). Dissolution of Cellulose in Ionic Liquid: A Review.

[33] Gao X., Shi Z., Liu C., Yang G., Sevostianov I., Silberschmidt V.V. (2015). Inelastic Behaviour of Bacterial Cellulose Hydrogel: In Aqua Cyclic Tests. Polym. Test..

[34] Astley O.M., Chanliaud E., Donald A.M., Gidley M.J. (2003). Tensile Deformation of Bacterial Cellulose Composites. Int. J. Biol. Macromol..

[35] Skvortsova Z.N., Gromovykh T.I., Grachev V.S., Traskin V.Y. (2019). Physicochemical Mechanics of Bacterial Cellulose. Colloid J..

[36] Chen S.-Q., Lopez-Sanchez P., Wang D., Mikkelsen D., Gidley M.J. (2018). Mechanical Properties of Bacterial Cellulose Synthesised by Diverse Strains of the Genus Komagataeibacter. Food Hydrocoll..

[37] Lee K.-Y., Tammelin T., Schulfter K., Kiiskinen H., Samela J., Bismarck A. (2012). High Performance Cellulose Nanocomposites: Comparing the Reinforcing Ability of Bacterial Cellulose and Nanofibrillated Cellulose. ACS Appl. Mater. Interfaces.

[38] Shezad O., Khan S., Khan T., Park J.K. (2010). Physicochemical and Mechanical Characterization of Bacterial Cellulose Produced with an Excellent Productivity in Static Conditions Using a Simple Fed-Batch Cultivation Strategy. Carbohydr. Polym..

[39] Bertasa M., Poli T., Riedo C., Tullio V.D., Capitani D., Proietti N., Canevali C., Sansonetti A., Scalarone D. (2018). A Study of Non-Bounded/Bounded Water and Water Mobility in Different Agar Gels. Microchem. J..

[40] Enev V., Sedláček P., Řihák M., Kalina M., Pekař M. (2022). IR-Supported Thermogravimetric Analysis of Water in Hydrogels. Front. Mater..

[41] Ul-Islam M., Khan T., Park J.K. (2012). Water Holding and Release Properties of Bacterial Cellulose Obtained by in Situ and Ex Situ Modification. Carbohydr. Polym..

[42] Swingler S., Gupta A., Gibson H., Kowalczuk M., Heaselgrave W., Radecka I. (2021). Recent Advances and Applications of Bacterial Cellulose in Biomedicine. Polymers.

[43] Ana R., Rebelo G.Y., Andrew J., Chen A.X., Liu C., Liu Y. (2018). Dehydration of Bacterial Cellulose and the Water Content Effects on Its Viscoelastic and Electrochemical Properties. Sci. Technol. Adv. Mater..

[44] Al-Emam E., Soenen H., Caen J., Janssens K. (2020). Characterization of Polyvinyl Alcohol-Borax/Agarose (PVA-B/AG) Double Network Hydrogel Utilized for the Cleaning of Works of Art. Herit. Sci..

[45] Coccato A., Bersani D., Coudray A., Sanyova J., Moens L., Vandenabeele P. (2016). Raman Spectroscopy of Green Minerals and Reaction Products with an Application in Cultural Heritage Research. J. Raman Spectrosc..

[46] Lafuente B., Downs R.T., Yang H., Stone N. (2015). The Power of Databases: The RRUFF Project. Highlights Mineral. Crystallogr..

[47] Szymańska-Chargot M., Cybulska J., Zdunek A. (2011). Sensing the Structural Differences in Cellulose from Apple and Bacterial Cell Wall Materials by Raman and FT-IR Spectroscopy. Sensors.

[48] Baglioni M., Poggi G., Chelazzi D., Baglioni P. (2021). Advanced Materials in Cultural Heritage Conservation. Molecules.

[49] Sbardella F., Pronti L., Santarelli M., Asua Gonzàlez J., Bracciale M. (2018). Waterborne Acrylate-Based Hybrid Coatings with Enhanced Resistance Properties on Stone Surfaces. Coatings.

[50] García O., Malaga K. (2012). Definition of the Procedure to Determine the Suitability and Durability of an Anti-Graffiti Product for Application on Cultural Heritage Porous Materials. J. Cult. Herit..

[51] Ortiz P., Antúnez V., Ortiz R., Martín J.M., Gómez M.A., Hortal A.R., Martínez-Haya B. (2013). Comparative Study of Pulsed Laser Cleaning Applied to Weathered Marble Surfaces. Appl. Surf. Sci..

[52] Becerra J., Mateo M., Ortiz P., Nicolás G., Zaderenko A.P. (2019). Evaluation of the Applicability of Nano-Biocide Treatments on Limestones Used in Cultural Heritage. J. Cult. Herit..

[53] Guilminot E. (2023). The Use of Hydrogels in the Treatment of Metal Cultural Heritage Objects. Gels.

[54] Pacelli S., Paolicelli P., Avitabile M., Varani G., Muzio L.D., Cesa S., Tirillò J., Bartuli C., Nardoni M., Petralito S. (2018). Design of a Tunable Nanocomposite Double Network Hydrogel Based on Gellan Gum for Drug Delivery Applications. Eur. Polym. J..

[55] Bakhtiari F., Darezereshki E. (2011). One-Step Synthesis of Tenorite (CuO) Nano-Particles from Cu4 (SO4) (OH) 6 by Direct Thermal-Decomposition Method. Mater. Lett..

[56] Schifano E., Cavallini D., De Bellis G., Bracciale M.P., Felici A.C., Santarelli M.L., Sarto M.S., Uccelletti D. (2020). Antibacterial Effect of Zinc Oxide-Based Nanomaterials on Environmental Biodeteriogens Affecting Historical Buildings. Nanomaterials.

