# Supplementary figures and images for: Bacterial Nanocellulose Hydrogel for the Green Cleaning of Copper Stains from Marble
Source: Gels. 2024 Feb 18;10(2):150. doi: 10.3390/gels10020150 (PMC10887925; doi:10.3390/gels10020150)

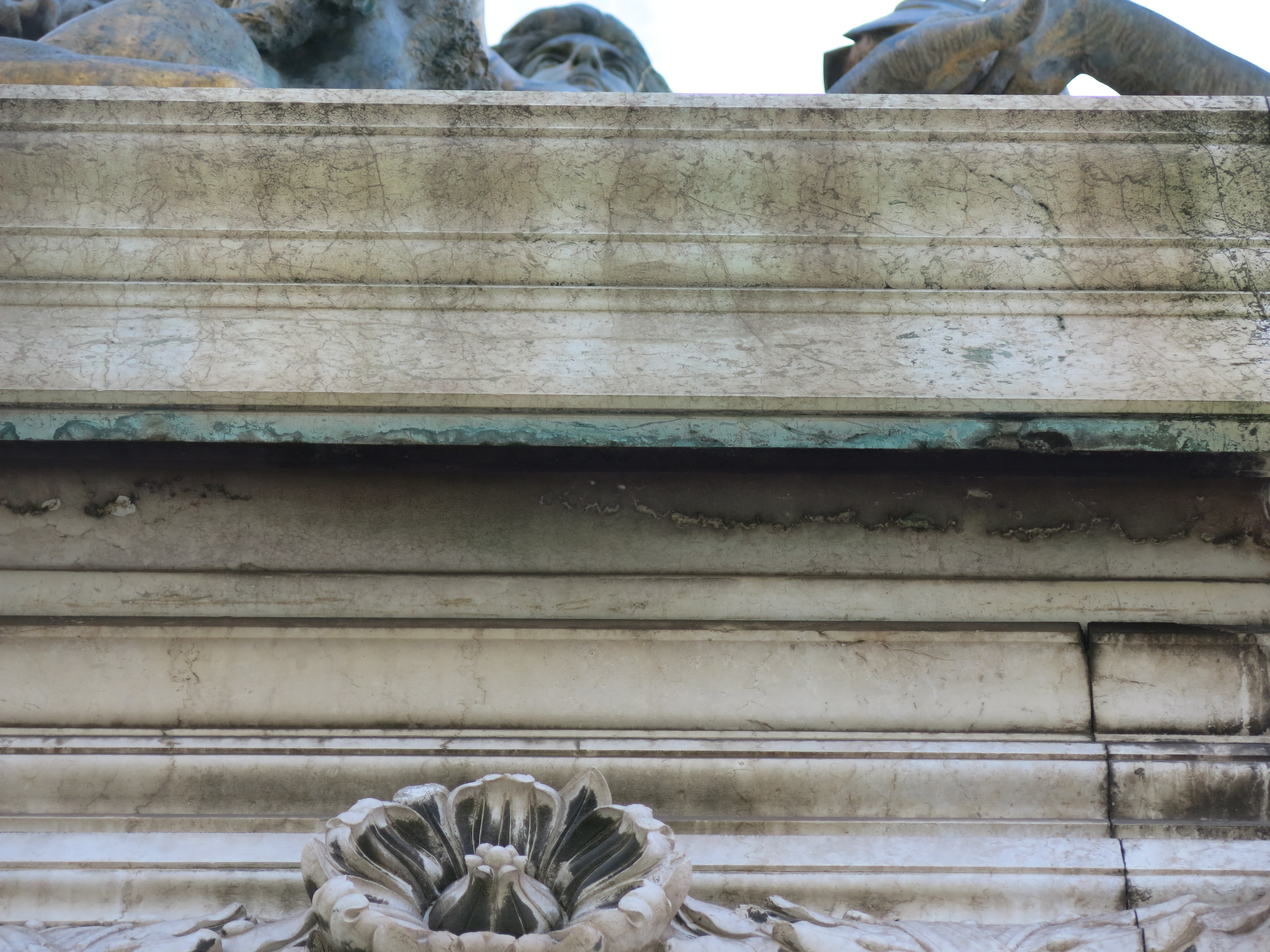

Supplement: Supplementary file 1 [file gels-10-00150-s001.zip › Figure S1.tif]

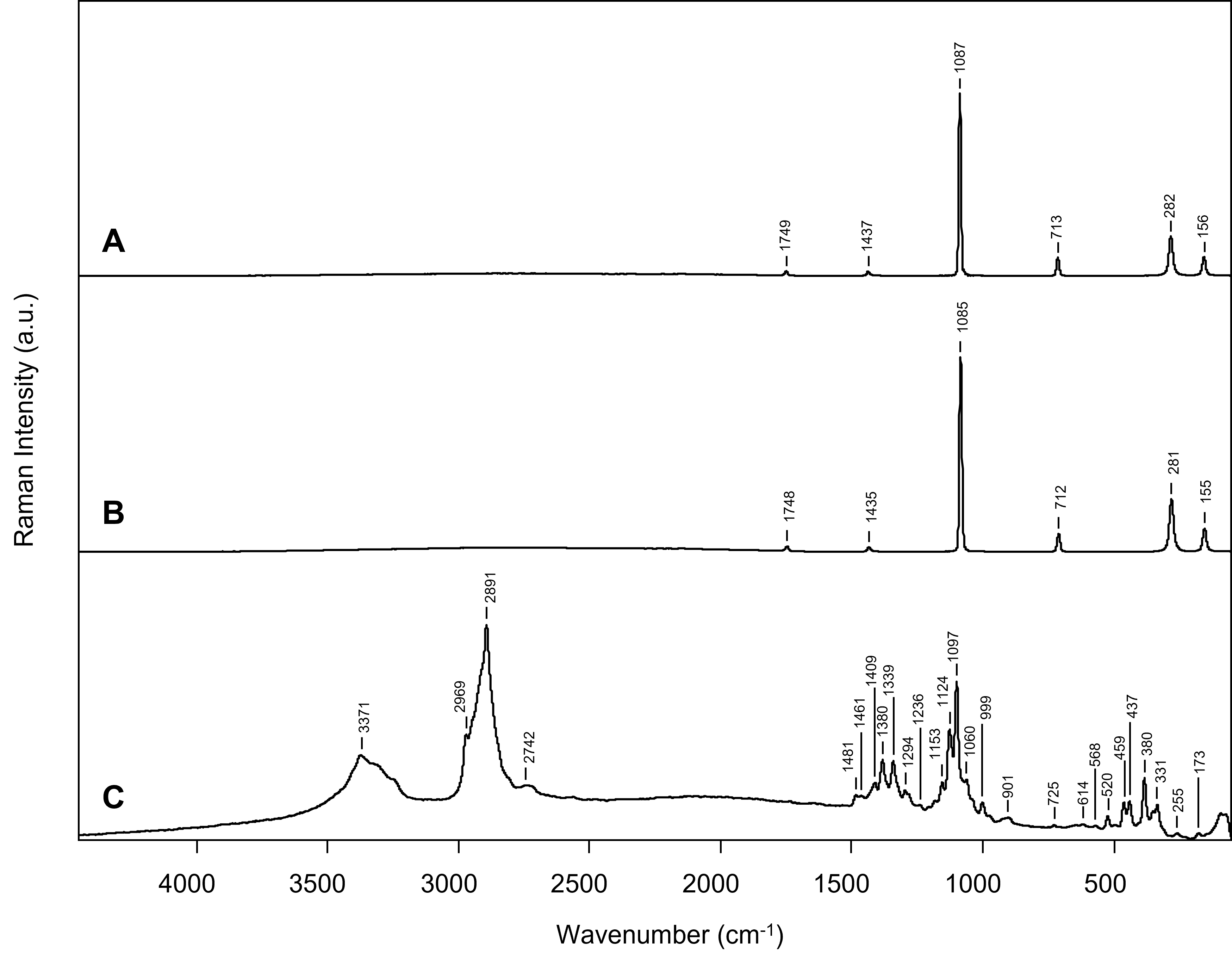

Supplement: Supplementary file 1 [file gels-10-00150-s001.zip › Figure S2.tif]

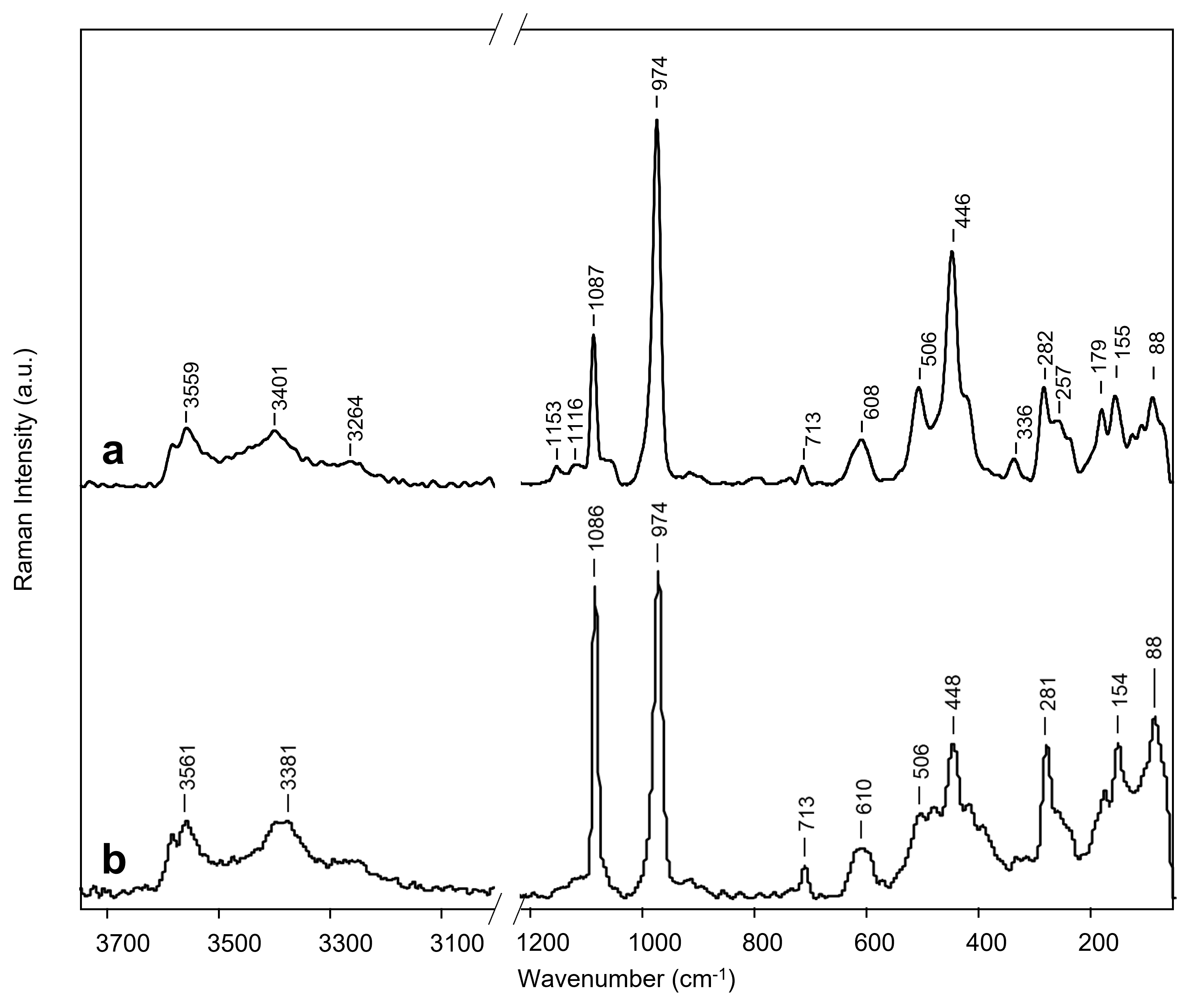

Supplement: Supplementary file 1 [file gels-10-00150-s001.zip › Figure S3.tif]

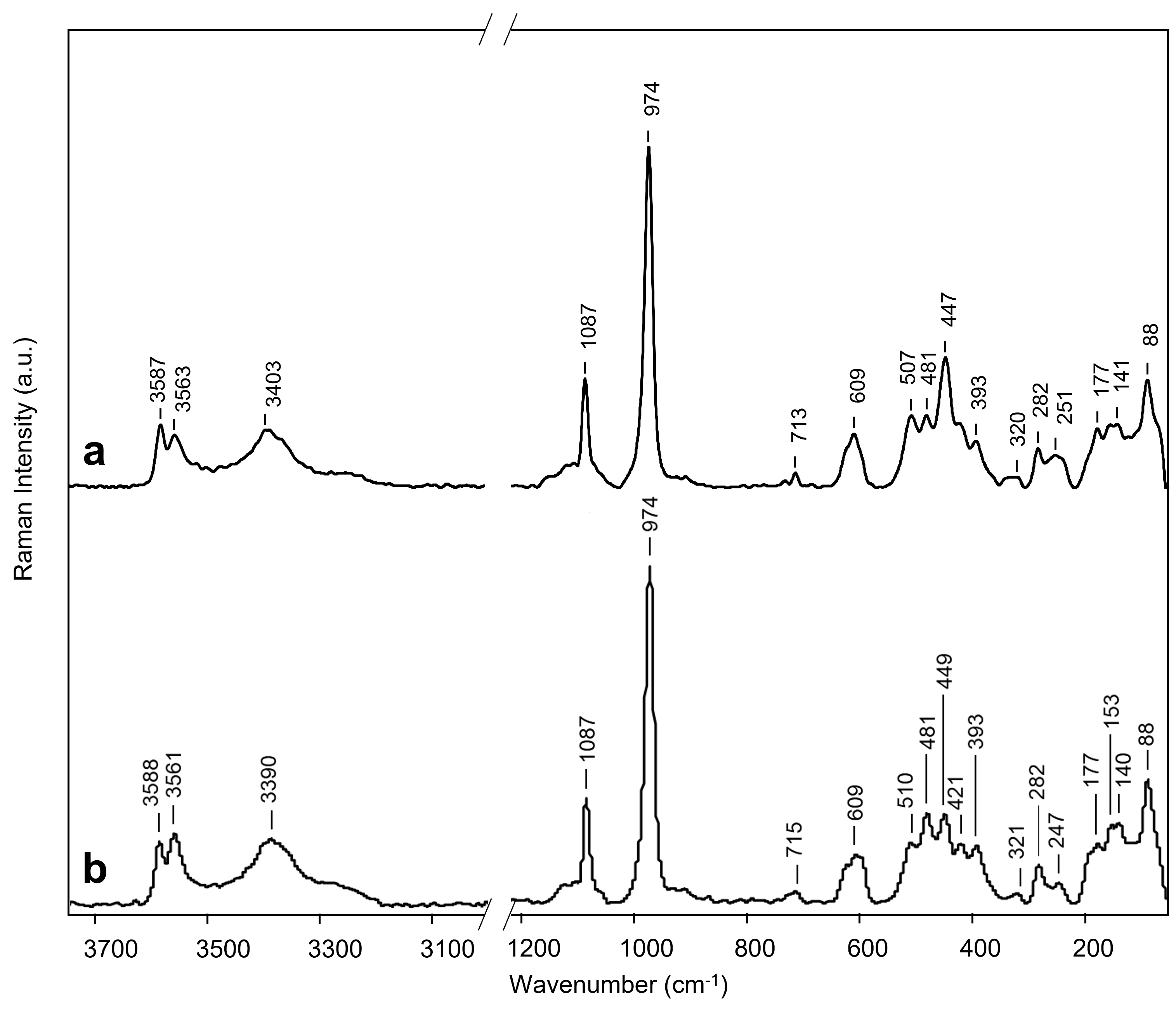

Supplement: Supplementary file 1 [file gels-10-00150-s001.zip › Figure S4.tif]
